# Validation of the Game Addiction Scale in Adolescents (GASA short version): adaptation for Mexican children

**DOI:** 10.1186/s41155-026-00393-2

**Published:** 2026-06-05

**Authors:** Enrique Romero-Pedraza, Roberto Lagunes-Córdoba, León Felipe Beltrán-Guerra, Erika Cortés-Flores

**Affiliations:** Present Address: Instituto de Investigaciones Psicológicas, Universidad Veracruzana, Xalapa, Mexico

**Keywords:** Video game addiction, addictive behavior, psychometric quality, reliability and validity.

## Abstract

**Objective:**

The objective of this study was to evaluate the psychometric quality of the Game Addiction Scale for Adolescents (GASA shortened version) in its adapted version for Mexican children.

**Methods:**

The instrument was administered to 289 primary school students using a non-probability quota sampling method. The average age of the participants was 10.47 years, with a standard deviation of 1.003 years, with 55% being boys and 45% being girls. Descriptive statistics were obtained from the seven items which make(made) up the scale to proceed with the confirmatory factor analysis (CFA), which was carried out using the robust estimation method *Satorra-Bentler* and the fit indices RMSEA (Root-mean square error of approximation), CFI (Comparative fit index), and SRMR (Standardized root mean square residual), as well as the X2/df ratio. Likewise Nevertheless, an analysis of the invariance of the factorial structure according to the sex of the participants was performed. Finally, reliability was determined by subscales using the McDonald’s omega coefficient.

**Results:**

The CFA shows a good adjustment data for the uni-factorial proposed model (X^2^/df = 1.26, *p* = .223; CFI = 0.992; RMSEA = 0.030, CI 90: 0.000 − 0.068; SRMR = 0.029). The Hoelter’s statistic was around 388.

**Conclusión:**

The post-COVID-19 context has created profitable conditions for the use or abuse of video games. The identification of addiction symptoms allows the strategies design of promotion and health intervention in vulnerable populations, such as the development of healthy games.

## Introduction

The internet increasingly adds in our daily lives and social environments, as well as the educational digitization, work, and spare time processes, has generated in a revolutionary way changes in the behaviour and consumption access in the diverse technologies and digital platforms.

In the context of the COVID-19 pandemic, where social distance was one of the measures to reduce infections, they also promoted behavioral changes, as well as in the way technologies were incorporated influencing daily life spaces in social, working, educational, and spare time activities (Hernández & Rivera, 2018; Del Prete & Redon, 2020; Han et al., 2022; Arellanez-Hernández et al., 2024).

In the children’s segment, the use of digital technologies has become an important tool at this developmental phase, as it offers a variety of possibilities for information access and learning acquisition, as well as socialization processes (Álvarez-García, et al, 2019).

It is particularly in the socialization integration processes related to digital contexts where the game works as a vehicle which enables social interaction, and therefore the *capacity development* of social bonds, creating networks in several social groups where the subject reveals video game traits, which differentiate from traditional (analog) games, making them much more attractive for children and young people (Arellanez-Hernández et al., 2024; Muros et al., 2013; Thorens et al., 2016).

The use of video games has become a way for having fun, spare-time, and even pastime from daily situations, as well as for reducing stress and anxiety, offering challenges, and the possibilities to remain in virtual spaces with constant and prompt rewards (inherent and external), with social reinforcements in complex narratives where the avatar can be played, which takes the player to an appearance condition, progression and frequency of symptoms which can be considered as a risk factor for mental health. Video games tend to be potentially addictive, especially in this population segment (Saunders et. al., 2017; Plante et al., 2019; Deleuze et al., 2019).

In 2019, the World Health Organization (OMS) warned about a disorder for video game-using, with symptoms which make reference to an addictive behavior, such as an uncontrollable need for playing, prioritizing the game over responsibilities, continue playing even no matter the negative consequences affecting personal areas as family, society, and job (World Health Organization, 2019; King & Delfabbro, 2020).

The International Classification of Diseases (ICD-11) places video game or digital use disorder(online or offline), as a disorder as a consequence of addictive behaviors, defining it as a recurrent behavior revealed in a lack of self-control when playing video games regarding time, frequency, intensity, duration and context. This behavior may occur as a marked distress consequence or degradation in the individual’s functional areas, whether it is continuously, episodically, or recurrently. Symptoms could occur in around a period of 12 months. (World Health Organization, 2021).

It is possible to identify studies that report a prevalence of video game use disorder globally of 3.3%, with a greater presence in the male population with a 8.5%, compared to the 3.5% presented in women, highlighting that such predominance is influenced by several factors such as the context and socio-demographic characteristics (Kim et al., 2022).

In terms of public health, the video game use disorder may become a problem in the mid-term if it is not prevented in time. To get this result, it is necessary to have tools and methodologies related to the specific population characteristics, which allow the strategy customization for a mental health promotion. (Darvesh et al., 2020)

The scales used for measuring video game addiction have gained relevance in recent years. However, until 2009 there were no validated scales to study specifically the video game addiction. This year, Lemmens has developed the Game Addiction Scale for Adolescents (GASA) based on seven DSM criteria for the diagnosis of game addiction: *Salience* refers to the fact that the video game acquires a dominant importance in a person’s life and controls thoughts, feelings, and behavior. *Tolerance* is an increase in the frequency and duration of play. *Excitement* is an euphoric or relaxing mood obtained as a result of gaming. *Withdrawal* refers to unpleasant emotions or physical effects experienced when gaming is suddenly stopped. *Relapse* is the tendency to return to gaming after a period of abstinence or reduction. *Conflict* refers to the deterioration of interpersonal relationships as a result of excessive gaming, including neglect and lying; and *problems* refer to impairment in social, academic, or work activities, as well as psychological disorders related to the loss of control over gaming behavior. (Lemmens et al., 2009).

Cross-validation research demonstrates that GASA, in its 7-items version, is a valid and reliable score to assess addictive issues about video games and since then, GASA has demonstrated validity to identify adolescents with problematic and abusive video games consumption.

As a psychometric measure, GASA has some advantages because is a brief 7-items score that can be administered quickly. Its validity and reliability had been reported similarly in several studies and different countries. Table 1 shows 19 validation studies of the GASA scale from 2014 to 2024. Ten were conducted in Europe, seven in Asia, and two in the Brazil. No validation of GASA has been found in American adolescents.

This review highlights efforts in Europe and Asia to adapt and validate these measurement instruments to assess addiction in their populations of adolescents, young adults, and adults, and the concern for understanding this phenomenon in different populations.

The reliability of these studies mostly reports Cronbach’s alpha values above 0.79, indicating good reliability. Regarding validity, the reported CFIs are generally high, up to 0.99, suggesting that the theoretical models of the scales have good validity in the different studied cultural contexts.

**Table 1:** Validations and adaptations of the GASA Scale in different countries

Author / Year	*N*	Study population	α	Ω	CFI	Country
(Gaetan et al., 2014)	306	Adolescents	0.79	-	0.97	France
(Brunborg et al., 2015)	10,081	Adults	-	-	0.96	Norway
(Lemos et al., 2015)	100	University students	0.92	-	-	Brasil
(Lemos et al., 2016)	384	University students	0.94	-	0.75	Brasil
(Baysak et al., 2016)	726	Adults	0.88	-	0.98	Turkey
(Khazaal et al., 2016)	3318	Adults	0.86	-	0.99	France
(Lloret-Irles et al., 2018)	466	Young people	0.81	-	0.95	Spain
(Khazaal et al. 2018a, b)	5983	Young people	0.99			Swiss
(Khazaal et al. 2018a, b)	2665	Adults	85	-	0.94	Germany
(Gaetan et al., 2014)	360	Adults	-	-	0.95	Indonesia
(Koga & Kawashima, 2018)	352	Adolescents	0.87	-	-	Japan
(Lin et al., 2019)	4,442	Adolescents	0.89	-	0.96	Irán
(Asaad et al., 2019)	50	Adolescents	0.81	-	-	Arabia
(Xu et al., 2019)	1067	Adults	-	-	0.9	China
(Liu et al., 2020)	1040	University students	0.95	0.96	0.99	China
(André et al., 2022)	144	Young people	-	-	0.99	Sweden
(Abolfotouh & Barnawi, 2024)	787	Adolescents	0.81	-	-	Arabia
(Roslan et al., 2024)	119	Young people	0.94		-	Malasia
(Tereshchenko & Gorbacheva, 2024)	2,380	Adolescents	0.81		-	Rusia
(Massano-Cardoso et al., 2024)	375	Adults	Adequate		-	Portugal

Studies on the video game addiction scales focused on children among the 8 to 12 years old are few or scarce. Most are conducted in populations over 14 years old. We assume this due to the digital divide, as children are at the time of these studies, not always they have the access to technological devices for gaming.

Studies found in teenagers among 12 and 17 years old, populations show that a 13.5% presented symptoms of gaming problems and a 3.3% a possible video game addiction when applying GASA in Spain. (Mora-Salgueiro et al., 2022). A research in Ecuador by Lucio and Ramos using another scale (Video Game Addiction Test) revealed that adolescents among 12 and 14 years old in their sample had significantly higher levels of addiction compared to those among 15 and 16 years old (Lucio & Ramos, 2024). In another study using the Internet Video Game Addiction Scale (IGD-20), being the subject a male adolescent, it was a significant predictor of problematic video game use in Ecuadorian (Ortega - Jiménez et al., 2023). A study in China to identify gender differences in problematic gaming among Chinese adolescents and young adults using the Video Game Dependence Scale found that men were more likely to engage in problematic video game use than women (21.5% vs. 14.1%), (Liao et al., 2025).

These studies show that video game addiction in adolescents has negative effects on their physical, mental, social, and academic health (Lliguipuma-Hidalgo et al., 2024), and this research, despite being scarce, shows that it could be similar in the case of children. A recent study used the Video Game-Related Experience Questionnaire to determine problematic use and understand the relationship between mobile device (MD)use and Mexican children. It was found that 41.6% of participants presented potential to serious problems with the video game and mobile device use (Martínez-Hernández et. al., 2025).

More research focused on children is needed to adequately capture the complexity of the phenomenon, emphasizing the importance of cultural adaptations to measurement tools. At the beginning of this research, we found only two specific scales of videogames addiction for children: the Children’s Video Game Addiction Questionnaire (CAVN), designed to assess addiction in children under 12 years old. (Mamani & Cuti, 2020). This scale, however, has 42 items, compared to the seven of the GASA score. The Video Game-Related Experience Questionnaire (VREQ) is a 17item scale that shows adequate psychometrical properties (Martínez-Hernández et al., 2025), but is twice more times longer than GASA and has less validity studies around the world.

Validating the GASA Scale in the Mexican population will provide an instrument suited to identifying symptoms related to video game addiction. Furthermore, early identification of addictive behaviors can lead children to create protective conditions which reduce the likelihood of developing addictive behaviors.

## Methods

### Design

This study was part of a more extended research to evaluate addictions in children and adolescents, including video games. As there was no scale to evaluate video game addiction available in Mexico, the GASA score was adapted to test its psycho-metrical properties and validity evidence in Mexican children under 12 years old.

### Participants

Two hundred eighty-nine students from Veracruz, Mexico, were recruited as non-probabilistic, convenience samples from fifth to sixth grade, from three, two elementary schools of Xalapa and one of Acatlan, Veracruz. In Xalapa, one was a public school, as the Acatlan one, and the other in Xalapa was private. Public schools students were of low socioeconomic standing and private school students were medium socioeconomic standing. 45% of this population were women and 55% men, with an average age among 10.47 and a standard deviation of 1.003 years.

Inclusion criteria were voluntary informed assent and informed consent signed by parents or legal tutors. Students under 8 years old or with health conditions which made difficult or impeded the understanding of GASA items were excluded. Incomplete records were eliminated from this analysis. Less than 5% of data were missed because of this reason.

### Instrument

*The Game Addiction Scale for Adolescents* (GASA-Short version) was used, in its validated Spanish seven-items version (Lloret-Irles et al., 2018). The scale consists of seven items that, through a five-point Likert-type measurement punctuated as follows: 1 = never, 2 = seldom, 3 = sometimes, 4 = frequently and 5 = always. The scale explores the frequency in which certain events associated with addictive behavior to video games occur or not: salience, tolerance, emotion, relapses, withdrawal, conflict and problems. Factor structure can be considered as uni-dimensional, as Lloret-Irles et al., (2018) found in their research, with a Cronbach alpha of 0.81 and 0.83 in two studies performed by these authors.

### Instrument adaptation

The scale was translated into Spanish directly from the English version for three bilingual researchers. A final one, consensus that translation was compared with Lloret-Irles et al., (2018) for another bilingual, independent researcher, finding it very similar and useful for the Mexican context. Content evaluation was performed by three experts: two in clinical psychology and one in psychometric measurement that suggested minimal sociocultural adaptations of each item. Particularly, the suggestion of beginning each item with the phrase “How frequently…”.

In the second stage, the scale was piloted with 30 students that found the scale as easy to understand and preferred the item phrase as our panel of judges suggested over the original by Lloret-Irles el al., (2018).

Finally, the instrument was applied to our working sample obtained by the procedure described in the next section.

### Procedure

Because the study’s objective was to validate the scale, several schools were contacted to seek their collaboration on the project. At first, principals from each school were contacted and given their approbation to perform the study. A presentation of the project was given to elementary school officials, explaining the objective, relevance, and importance of having an instrument that would screen children for video game use.

After agreeing to participate in the project, each school held an informational meeting for students and parents to invite them to participate in the study. Informed assent and consent were explained, resolving all the doubts that parents and students expressed. Researchers took great care to emphasize the voluntary participation in the study, the right of rejection to continue for any cause, and that negative for participating had no consequences in grades or scholar issues of students.

The instrument, as part of a wider battery of tests, was administered in groups at each school by the research team, taking approximately 10 min to fill all formats. No student expressed discomfort with the scale’s content.

### Ethical considerations

For the design and execution of the project, internationally established ethical standards were considered, such as the Declaration of Helsinki (World Medical Association [WMA], 2013), as well as current Mexican regulations applicable to the conduct of research on humans (Secretaría de Salud, 1983), such as the General Health Law Regulations and the Mexican Official Standard, Nom028 (Secretaría de Salud, 2013), prioritizing the physical and emotional integrity of the participants and guaranteeing their anonymity and confidentiality through informed consent for the parents of the children who agreed to participate.

This study was part of a more comprehensive intervention named “¿Qué pasa si te pasas?”, with federal and state collaborations. All procedures, including the GASA score application, were approved by ethics board of the “Instituto Nacional de Psiquiatría Ramón de la Fuente Muñiz” with the register number CEI/C/003/2016. All parents of children received and signed the informed consent. For children, informed assent was also offered on the day of the instrument administration. The Federal Law on the Protection of Personal Data Held by Private Parties (2010) was also considered, due to its regulations that establish the need to keep minors’ data private.

### Statistical analysis

Descriptive statistics were obtained for the seven items comprising the scale. Since a uni-dimensional factor structure was already corroborated in adolescents (Lloret-Irles et al., 2018), confirmatory factor analysis (CFA) was used for this study.

Firstly, it was contrasted to the normality uni and multi multivariate using the Shapiro-Wilk test and the Mardia test, respectively. The discriminant power of the items was then analyzed by comparing scores in the first quartile with those in the fourth quartile using the Mann-Whitney U test (Bandalos, 2018).

As a priori, probabilistic sample was not feasible for this study, a post-hoc power analysis was performed to test if statistical power is adequate (above 80%) to detect an RMSEA fit index of maximum 0.08 with the R package semPower version 2.1.3 (Moshagen & Bader, 2025).

Since no type of normality was found (Shapiro-Wilk, *p*<.001 for the seven items; Mardia test, *p*=.015), the CFA was carried out using the Satorra-Bentler robust estimation method and the RMSEA ( *Root-mean square error of approximation* ), CFI ( *Comparative fit index* ) and SRMR ( *Standardized root mean square residual* ) fit indices were used, as well as the X ^2^ /df ratio, in accordance to the criteria in force in the literature on structural equations (Kline, 2023).

The invariance of the factorial structure was determined according to the sex of the participants, taking under consideration the studies which have identified sex differences in study populations with similar characteristics (Arellanez -Hernández et al., 2024), as with the GASA score in adolescents (Gaetan et al., 2014; Lloret-Irles et al., 2018; Liu et al., 2020). The analyses were carried out following current psychometric criteria (Fischer & Karl, 2019; Svetina et al., 2020), through a sequential process in which differences between groups are evaluated, in the configuration of the instrument (configural invariance), the equivalence of the factorial loads (metric invariance), the intercepts (strong invariance), and the residuals between the groups (strict invariance).

Finally, also due to the lack of normality of the data, reliability was determined by subscales using the McDonald’s omega coefficient. The descriptive analysis of normality, discriminative power and McDonald’s omega coefficient analyses. The CFA and the invariance measures were performed with the JASP program, version 0.95.2. The invariance analysis was performed using the *lavaan package* of the R *software* (Rosseel, 2025).

## Results

Table 2 summarizes the descriptive statistics for the seven items in the scale. All items had adequate discriminatory power and a positive correlation superior to 0.60 with the average scale; which is why they were retained for realizing the confirmatory factorial analysis.

**Table 2:** Descriptive statistics of the GASA scale items (*n* = 289)

Item	Average (SD)	Skewness	Kurtosis	*r* reactive-scale*	Shapiro-Wilk (*p*)
Do you think about playing during the day?	2.79 (1.19)	0.11	-0.77	0.699	< 0.001
Have you increased the amount of time you spend playing?	3.43 (1.3)	− 0.29	-1.03	0.687	< 0.001
Do you play to forget about real life?	3.44 (1.38)	− 0.41	-1.04	0.652	< 0.001
Have other people tried to make you reduce the time you spend playing?	3.06 (1.5)	− 0.10	-1.39	0.609	< 0.001
Have you ever felt bad when you haven’t been able to play?	3.68 (1.3)	− 0.60	− 0.81	0.710	< 0.001
Have you ever argued with others (friends, siblings, parents…) about the amount of time you spend playing?	4.13 (1.18)	-1.30	− 0.77	0.618	< 0.001
Have you left unattended important activities (studies, family, sports) to play?	3.87 (1.34)	− 0.92	− 0.41	0.672	< 0.001

*.- Spearman correlation coefficient

All items have adequate discriminatory power and a positive correlation greater than 0.60 with the total scale, which is why they have all been retained to perform the confirmatory factorial analysis.

**Fig. 1:**
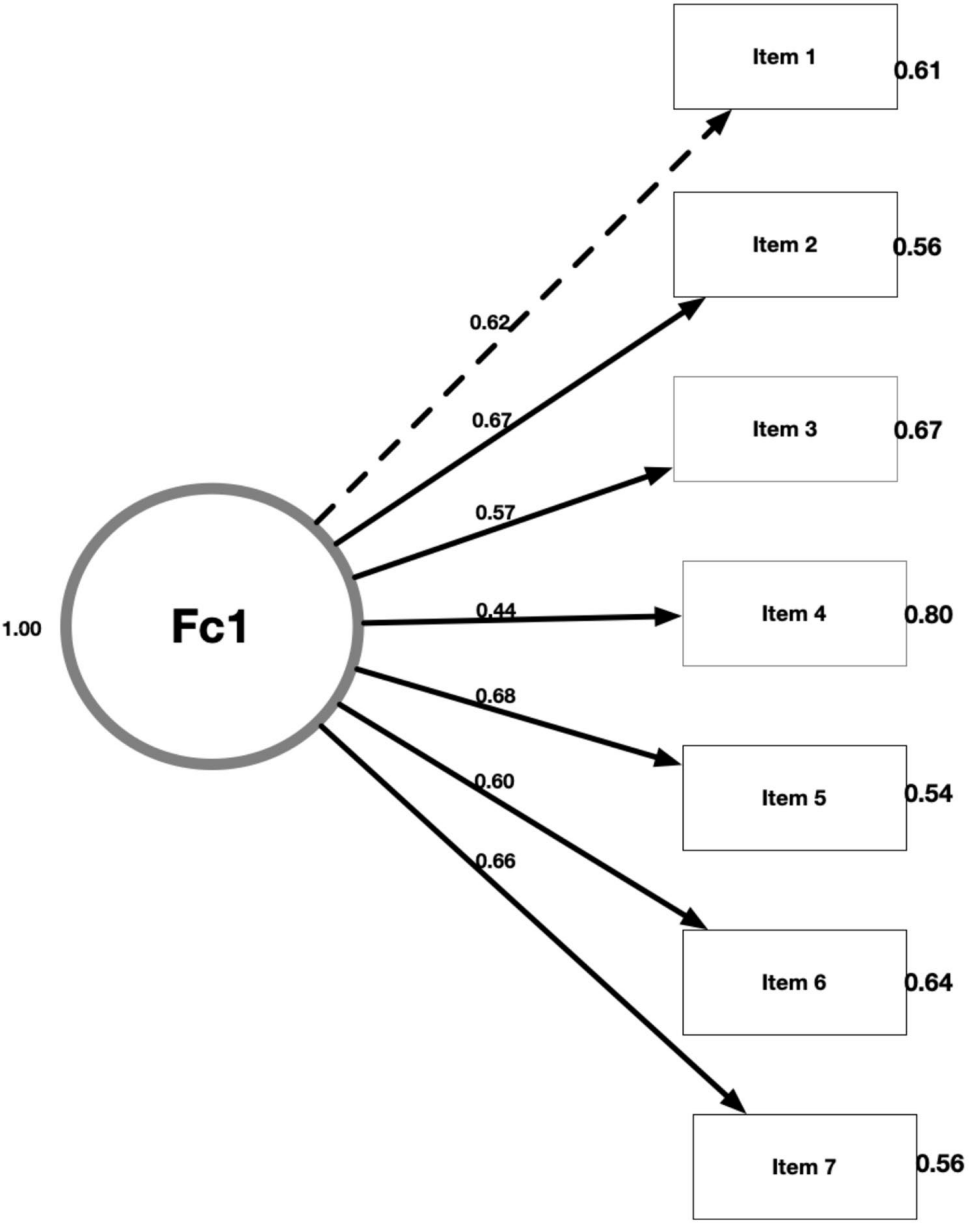
Factorial structure of the GASA scale in primary school children. Factor loading and error variances are shown. Items are labeled in order with respect to Table 1. Fc1: game addiction total score. Items are labeled in order as Table 1

Post-hoc power analysis showed that statistical power for the sample (*n* = 289), with 14 degrees of freedom, has a statistical power of 93.6% to detect a RMSEA fit index of 0.08. The CFA shows a very good fit of the data to the proposed univariate model (X ^2^ /df = 1.26, *p* = .223; CFI = 0.992; RMSEA = 0.030, CI 90: 0.000 − 0.068; SRMR = 0.029). The Hoelter statistic was 388, and the average variance extracted was 0.366 (Fig. 1).

As it is shown in Table 3, Invariance analysis showed that the structure remains invariant for both sexes even when the measures, intercepts and residuals are restricted.

The reliability analysis showed an acceptable McDonald’s index (ω = 0.80).

Table 4 shows that there exist because of the sex differences there are in responses to some of the test items. Boys have higher scores on all items, with the exception of the game which makes them forget about real-life problems. Nevertheless, the differences should be interpreted with caution, as the effect size in all cases is small.

Table 5 shows the items of the GASA score, Mexican version.

**Table 3:** Adjustment indices and invariance assessment by Sex

	CFI	RMSEA (CI 90%)	SRMR	ΔCFI	ΔRMSEA	ΔSRMR
Configural	0.977	0.050 (0.000-0.087)	0.044			
Metric	0.978	0.045 (0.000-0.079)	0.059	0.001	− 0.005	0.015
Scalar	0.973	0.046 (0.000-0.078)	0.059	− 0.005	0.001	0.000
Strict	0.974	0.041 (0.000-0.072)	0.063	0.001	− 0.005	0.004

**Table 4:** Results by gender in the GASA score

Item	Average Range Boys	Average Range Girls		P*	Effect size (*r*)
Do you think about playing during the day?	165.5		119.8	< 0.001	0.31
Have you increased the amount of time you spend playing?	152.8		135.4	0.002	0.21
Do you play to forget about real life?	157.2		130.1	0.070	0.12
Have other people tried to get you to cut down on your gaming time?	155.6		132.1	0.005	0.18
Have you felt bad when you couldn’t play?	156.3		131.2	0.013	0.16
Have you ever argued with others (friends, siblings, parents…) about the amount of time you spend playing?	155.8		131.7	0.009	0.17
Have you neglected important activities (studies, family, sports) to play?	163.8		121.9	< 0.001	0.16

*.- Mann-Whitney U test

**Table 5:** GASA score, Mexican version

Item
¿Con qué frecuencia piensas en jugar durante el día?
¿Has aumentado el tiempo que dedicas a jugar?
¿Qué tan frecuentemente juegas para olvidarte de la vida real?
¿Con qué frecuencia otras personas han intentado que reduzcas el tiempo que dedicas al juego?
¿Con qué frecuencia te has sentido mal cuando no has podido jugar?
¿Con que frecuencia te has peleado con otros (amigos, padres, ) por el tiempo que dedicas al juego?
¿Qué tan frecuentemente te has desatendido actividades importantes (estudios, familia, deportes) por jugar?

## Discussion

The seven-item structure of the GASA score, Mexican version, is the same as the original in Lemens et al. (2009), and the Spanish adaptation by Lloret-Irles et al. (2018). Factor loadings were not reported in Lemmens et al., (2009) for the seven items version, but reported ones in Spanish version are very similar to the Mexican, with factor loadings ranging from 0.48 to 0.74 and Cronbach’s alpha of 0.81. This is very similar to other studies that confirmed the seven-item structure for GASA in adolescents, as Costa et al., 2020; Liu et al., 2020; and Andre et al., 2022.

This finding shows evidence that seven-item GASA score can be culturally adapted retaining structural and psychometrical properties, not only for adolescents, but even for children from 8 to 12 years old. Of course, more international validation studies must be performed before it can be stated, but the scale fitting to unidimensional models and stable factor loadings across studies could make valuable future studies for validation in other countries.

The item wording of the Mexican GASA version is very similar to Loret-Irles et al., 2018 for Spanish GASA score in adolescents. However, there is a noticeable difference. In the pilot study, we found that it is easier for children to understand the Likert scale if all the items begin with the text “How frequently… (*Con qué frecuencia*)”. This, which can be repetitive or disturbing for a long scale for other age groups, was clearer and easier to understand for children in our sample. Research has shown that format and response options for Likert scores in children affects reliability and item understanding (Mellor & Moore, 2014; Alan & Kabasakal, 2020). So, this is an issue that must be assessed in every validation study.

The average variance extracted (AVE) measures the amount of variance that a latent construct captures from its indicators relative to the variance due to measurement error. Fornell and Larcker (1981) proposed a minimum AVE = 0.050 to assess if a scale has an adequate convergent validity. In our sample, GASA score has an AVE of 0.366, that is non adequate based on Fornell and Larcker criteria. However, De la Rubia (2019), in his revision of AVE criteria, has stated that AVE > 0.50 is acceptable when composed reliability, factor loadings and item numbers meet certain conditions. According to Table 1 in De la Rubia (2019), for seven items and McDonald omega = 0.80, an AVE = 0.364 and a medium standardized factor loading of 0.603 is required for an acceptable convergent validity. In our sample, AVE was 0.366 and medium standardized factor loading is 0.605 (determined as a medium of factor loadings), meeting the criteria stated in De la Rubia revision.

As stated early, there are at least two other scales to assess videogame addiction in children: the Children’s Video Game Addiction Questionnaire (CAVN), designed to assess addiction in children under 12 years of age (Mamani & Cuti, 2020), and The Video Game-Related Experience Questionnaire (VREQ, Martínez-Hernández et al., 2025). These scales are not only considerably longer than GASA, but they have a more complicated factor structure (five factors for CAVN and two factors for VREQ). In general, a simpler structure is more replicable across samples (Ferrando & Anguiano-Carrasco, 2010), and it seems to be the case for GASA score for adolescents. If future studies in children have similar results, GASA could be used for multinational and transcultural studies about game addiction. Additionally, the length of GASA is less than a half of VREQ and CAVN, making this more acceptable for children and easier to include as part of a battery of tests.

As technologies are nowadays more present in everyday life, they could favor certain conditions of vulnerability to trigger addictive behavior to video games, as can be seen in the various studies identified that address this problem (Kim et al., 2022; Liao et al., 2025; Lliguipuma-Hidalgo et al., 2024; Martínez-Hernández et al., 2025; Ortega - Jiménez et al., 2023). Due to its reliability and psychometrical properties, the use of GASA will provide valuable information for evaluating videogame addiction, making it suitable in diagnostic and intervention studies, designing health promotion and prevention strategies for healthy behaviors about videogames in children.

Results of this study must be interpreted based on its limitations. In spite of psychometrical properties of GASA are sound, the use of a non-probabilistic sample limits its generalizability to other samples or contexts, even in Mexican children samples. However, due to the fact that the factor structure and factor loadings are similar to the findings in literature, it is possible that these can be replicated in future studies with larger and representative samples. Important evidence of this was the result of post-hoc power analysis. Sample power to detect acceptable, sound RMSEA of 0.08 was superior to 90%, indicating high probability that scale fitting to one-factor structure is not spurious.

Invariance of GASA in children samples is an issue that needs more research. We investigated gender invariance because several reports found only metric and configural invariance, with only partial strict invariance. In our sample, we found evidence for strong and strict invariance, as changes in CFI, RMSEA and SRMR are not enough to meet criteria of Svetina et al. (2020). This finding is interesting, because there are statistical differences between scores in boys and girls for all items, except number three. However, in spite of their statistical significance, the effect size is small in all cases. It is possible that the sample lacked statistical power to detect small differences in means and intercepts. This issue needs more research with a bigger sample before it can be stated that GASA scores are invariant for gender in children under 12 years old.

Future research with the GASA score could include replication studies in larger, more representative samples, as validity evidences need to be always population and context adapted (Bandalos, 2018). The score can be used to diagnose, assess, and test interventions to diminish the use of videogames and its associated negative effects. Its unidimensional structure is well suited to transcultural and multinational studies.

## Data Availability

The original contributions presented in the study are included in the article/supplementary material, further inquiries can be directed to the corresponding authors.

## References

[CR1] Abolfotouh, M. A., & Barnawi, N. A. (2024). Prevalence and Prediction of Video Gaming Addiction Among Saudi Adolescents, Using the Game Addiction Scale for Adolescents (GASA). *Psychology Research and Behavior Management*, *17*, 3889–3903. 10.2147/PRBM.S493776 39559711 10.2147/PRBM.S493776 PMC11570534

[CR2] Alan, Ü., & Kabasakal, K. A. (2020). Effect of number of response options on the psychometric properties of Likert-type scales used with children. *Studies in Educational Evaluation*, *66*, 100895. 10.1016/j.stueduc.2020.100895

[CR3] Álvarez-García, D., García, T., Cueli, M., & Núñez, J. C. (2019). [Control Parental del Uso de Internet durante la Adolescencia: Evolución y Diferencias de Género]. *Revista Iberoamericana de Diagnóstico y Evaluación - e Avaliação Psicológica. RIDEP*. 2(51). https://doi.org10.21865/RIDEP51.2.02.

[CR4] André, F., Munck, I., Håkansson, A., & Claesdotter-Knutsson, E. (2022). Game Addiction Scale for Adolescents—Psychometric Analyses of Gaming Behavior, Gender Differences and ADHD. *Frontiers in Psychiatry*, *13*, 791254. 10.3389/fpsyt.2022.791254 35356720 10.3389/fpsyt.2022.791254 PMC8959768

[CR5] Arellanez-Hernández, J. L., Romero-Pedraza, E., Beltrán-Guerra, L. F., & Lima-Zarate, F. I. (2024). [Comparación de características adictivas a los videojuegos entre hombres y mujeres estudiantes mexicanos]. *Revista Psicología y Salud*, *34*(1), 135–144. 10.25009/pys.v34i1.2851

[CR6] Asaad, T., Morsy, K. E., Hasan, H., Din, E., M. H., & Meguid, E., M. A (2019). Game Addiction Scale for Adolescents: Arabic Version Validation. *Addictive Disorders and their Treatment*, *18*(4), 223–228. 10.1097/ADT.0000000000000167

[CR7] Bandalos, D. L. (2018). *Measurement Theory and Applications for the Social Sciences*. The Guilford Press.

[CR8] Baysak, E., Duygu-Kaya, F., Dalgar, I., & Candansayar, S. (2016). Online Game Addiction in a Sample from Turkey: Development and Validation of the Turkish Version of Game Addiction Scale. *Bulletin of Clinical Psychopharmacology*, *26*(1), 21–31. 10.5455/bcp.20150502073016

[CR9] Brunborg, G. S., Hanss, D., Mentzoni, R. A., & Pallesen, S. (2015). Core and Peripheral Criteria of Video Game Addiction in the Game Addiction Scale for Adolescents. *Cyberpsychology Behavior and Social Networking*, *18*(5), 280–285. 10.1089/cyber.2014.0509 25826043 10.1089/cyber.2014.0509 PMC4432774

[CR10] Costa, S., Barberis, N., Gugliandolo, M. C., Liga, F., Cuzzocrea, F., & Verrasto, V. (2020). Examination of the Psychometric Characteristics of the Italian Version of the Game Addiction Scale for Adolescents. *Psychological Reports*, *124*(3), 1365–1381. 10.1177/0033294119838758 10.1177/0033294119838758 30940015

[CR11] Darvesh, N., Radhakrishnan, A., Lachance, C., Nincic, V., Sharpe, J., Ghassemi, M., Straus, S., & Tricco, A. (2020). Exploring the prevalence of gaming disorder and Internet gaming disorder: a rapid scoping review. *Syst Rev*, *9*, 68. 10.1186/s13643-020-01329-2 32241295 10.1186/s13643-020-01329-2 PMC7119162

[CR12] Del Prete, A., & Redon Pantoja, S. (2020). [Las redes sociales on-line: Espacios de socialización y definición de identidad]. *Psicoperspectivas*, *19*(1), 1–11. 10.5027/psicoperspectivas-vol19-issue1-fulltext-1834

[CR13] Deleuze, J., Maurage, P., Schimmenti, A., Nuyens, F., Melzer, A., & Billieux, J. (2019). Escaping reality through videogames is linked to an implicit preference for virtual over real-life stimuli. *Journal Of Affective Disorders*, *15*(245), 1024–1031. 10.1016/j.jad.2018.11.078 10.1016/j.jad.2018.11.078 30699844

[CR14] Ferrando, J. P., & Anguiano-Carrasco, C. (2010). [El análisis factorial como técnica de Investigación en psicología]. *Papeles del Psicólogo*, *31*(1), 18–33. https://www.redalyc.org/pdf/778/77812441003.pdf

[CR15] Fischer, R., & Karl, J. A. (2019). A Primer to (Cross-Cultural) Multi-Group Invariance Testing Possibilities in R. *Frontiers in Psychology*, *10*, 1–18. 10.3389/fpsyg.2019.01507 31379641 10.3389/fpsyg.2019.01507 PMC6657455

[CR16] Fornell, C., & Larcker, D. F. (1981). Evaluating structural equation models with unobservable variables and measurement error. *Journal of Marketing Research*, *18*(1), 39–50. 10.2307/3151312

[CR17] Gaetan, S., Bonnet, A., Brejard, V., & Cury, F. (2014). French validation of the 7-item Game Addiction Scale for adolescents. *Revue europeenne de psychologie appliquee*, *64*(4), 161–168. 10.1016/j.erap.2014.04.004

[CR18] Han, T., Cho, H., Sung, D., & Park, M. (2022). A systematic review of the impact of COVID-19 on the game addiction of children and adolescents. *Frontiers in Psychiatry*, *13*. 10.3389/fpsyt.2022.976601 10.3389/fpsyt.2022.976601 PMC9435970 36061296

[CR19] Hernández Contreras, C., & Rivera Ottenberger, D. (2018). [Adaptación Transcultural y Evaluación de las Estructuras Factoriales del Test de Adicción a Internet en Chile: Desarrollo de una Versión Abreviada]. *Revista Iberoamericana de Diagnóstico y Evaluación - e Avaliação Psicológica. RIDEP*. 4(49), 143–155. 10.21865/RIDEP49.4.12

[CR21] Khazaal, Y., Chatton, A., Rothen, S., Achab, S., Thorens, G., Zullino, D., & Gmel, G. (2016). Psychometric properties of the 7-item game addiction scale among french and German speaking adults. *Bmc Psychiatry*, *16*(1). 10.1186/s12888-016-0836-3 10.1186/s12888-016-0836-3 PMC4862221 27160387

[CR20] Khazaal, Y., Breivik, K., Billieux, J., Zullino, D., Thorens, G., Achab, S., Gmel, G., & Chatton, A. (2018a). Game addiction scale assessment through a nationally representative sample of young adult men: Item response theory graded-response modeling. *Journal of Medical Internet Research*, *20*(8). 10.2196/10058. Scopus. 10.2196/10058 PMC6131318 30150204

[CR22] Khazaal, Y., Breivik, K., Billieux, J., Zullino, D., Thorens, G., Achab, S., Gmel, G., & Chatton, A. (2018b). Game Addiction Scale Assessment Through a Nationally Representative Sample of Young Adult Men: Item Response Theory Graded–Response Modeling. *Journal of Medical Internet Research*, *20*(8), e10058. 10.2196/10058 30150204 10.2196/10058 PMC6131318

[CR23] Kim, H. S., Son, G., Roh, E. B., Ahn, W. Y., Kim, J., Shin, S. H., Chey, J., & Choi, K. H. (2022). Prevalence of gaming disorder: A meta-analysis. *Addictive behaviors*. 126. 10.1016/j.addbeh.2021.107183 10.1016/j.addbeh.2021.107183 34864436

[CR24] King, D. L., & Delfabbro, P. H. (2020). Chapter 7 - Video game addiction. In Cecilia A. Essau, Paul H. Delfabbro (Eds.), In *Practical Resources for the Mental Health Professional, Adolescent Addiction* (Second Edition), Academic Press, pp. 185–213.

[CR25] Kline, R. E. (2023). *Principles and practice of structural equation modelling* (5–(5.ͣ.). ed.). The Guilford Press.

[CR26] Koga, Y., & Kawashima, D. (2018). Development and validation of japanese version of the game addiction scale for adolescents. *The Japanese Journal of Personality*, *27*(2), 175–177. 10.2132/personality.27.2.10

[CR27] Lemmens, J. S., Valkenburg, P. M., & Peter, J. (2009). Development and Validation of a Game Addiction Scale for Adolescents. *Media Psychology*, *12*(1), 77–95. 10.1080/15213260802669458

[CR29] Lemos, I. L., Conti, M. A., & Sougey, E. B. (2015). Evaluation of semantic equivalence and internal consistency of the game addiction scale (GAS): Portuguese version. *Jornal Brasileiro de Psiquiatria*, *64*(1), 8–16. 10.1590/0047-2085000000051

[CR28] Lemos, I. L., Cardoso, A., & Sougey, E. B. (2016). Validity and reliability assessment of the Brazilian version of the game addiction scale (GAS). *Comprehensive Psychiatry*, *67*, 19–25. 10.1016/j.comppsych.2016.01.014. Scopus. 27095330 10.1016/j.comppsych.2016.01.014

[CR30] Liao, Z., Le, J., Chen, X., Tang, Y., Shen, H., & Huang, Q. (2025). Gender differences in problematic gaming among Chinese adolescents and young adults. *Bmc Psychiatry*, *25*(1), 522. 10.1186/s12888-025-06994-y 40405153 10.1186/s12888-025-06994-y PMC12096608

[CR31] Lin, C. Y., Imani, V., Broström, A., Årestedt, K., Pakpour, A. H., & Griffiths, M. D. (2019). Evaluating the Psychometric Properties of the 7-Item Persian Game Addiction Scale for Iranian Adolescents. *Frontiers in Psychology*, 10. 10.3389/fpsyg.2019.00149 10.3389/fpsyg.2019.00149 PMC6370725 30804841

[CR32] Liu, Y., Wang, Q., Jou, M., Wang, B., An, Y., & Li, Z. (2020). Psychometric properties and measurement invariance of the 7-item game addiction scale (Gas) among Chinese college students. *Bmc Psychiatry*, *20*(1), 484. 10.1186/s12888-020-02830-7 33008339 10.1186/s12888-020-02830-7 PMC7531159

[CR33] Lliguipuma-Hidalgo, L. L., Yaucan-Mero, J. G., Medina-Ruela, K. P., & Albán-Sabando, E. A. (2024). [Efectos Nocivos De La Adicción a Los Videojuegos en Los Adolescentes]. *Mqrinvestigar*, *8*(3), 3613–3623. 10.56048/mqr20225.8.3.2024.3613-3623

[CR34] Lloret-Irles, D., Morell-Gomis, R., Marzo, J. C., & Tirado, S. (2018). [Validación española de la Escala de Adicción a Videojuegos para Adolescentes – GASA]. *Atención Primaria*, *50*(6), 350–358. 10.1016/j.aprim.2017.03.015 28939247 10.1016/j.aprim.2017.03.015 PMC6836933

[CR35] Lucio, N. D. T., & Ramos, D. C. G. (2024). [Hostilidad Y Dependencia a Videojuegos en Adolescentes]. *Revista Científica Arbitrada Multidisciplinaria Pentaciencias*, *6*(4), 210–220. 10.59169/pentaciencias.v6i4.1130

[CR36] Mamani, Y. B., & Cuti, M. C. (2020). [Construcción Y Propiedades Psicométricas De Un Cuestionario Para Medir El Nivel De Adicción a Videojuegos en Red (CUAVIR) De La Ciudad De Juliaca – 2020]. *Revista Científica De Ciencias De La Salud*, *13*(2), 9–19. 10.17162/rccs.v13i2.1423

[CR37] Martínez-Hernández, R., Zamarripa, J., & Rocha, G. M. N. (2025). Psychometric Properties of the Video Game Experiences Questionnaire (CERV), Problematic Use of Video Games and the Link with the Use of Mobile Devices in Mexican Children. *International Journal of Environmental Research and Public Health*, *22*(4). 10.3390/ijerph22040476 10.3390/ijerph22040476 PMC12026956 40283705

[CR38] Massano-Cardoso, I., Nogueira, F., Carvalho-Figueiredo, S., & Galhardo, A. (2024). European Portuguese Version of the Game Addiction Scale-7: Factor Structure and Psychometric Properties. *European Addiction Research*, *30*(4), 1–7. 10.1159/000539712 38986467 10.1159/000539712

[CR39] Mellor, D. M., & Moore, K. A. (2014). The Use of Likert Scales With Children. *Journal of Pediatric Psychology*, *39*(3), 369–379. 10.1093/jpepsy/jst079 24163438 10.1093/jpepsy/jst079

[CR40] Mora-Salgueiro, J., Feijóo, S., Tobío, T. B., Mallou, J. V., & Boubeta, A. R. (2022). [Hábitos De Juego Y Síntomas De Adicción a Los Videojuegos en Adolescentes Españoles]. *Behavioral Psychology/Psicología Conductual*, *30*(3), 627–639. 10.51668/bp.8322302s

[CR41] Moral-de la Rubia, J. (2019). Revisión de los criterios para validez convergente estimada a través de la Varianza Media Extraída. *Psychologia*, *13*(2), 25–41. 10.21500/19002386.4119

[CR42] Moshagen, M., & Bader, M. (2025). Package ‘semPower’. Power analyses for SEM. CRAN repository. https://github.com/moshagen/semPower

[CR43] Mujiya Ulkhaq, M., Rozaq, R., Ramadhani, R., Heldianti, R., Fajri, A., & Akshinta, P. Y. (2018). Validity and reliability assessment of the Game Addiction scale: An empirical finding from Indonesia. *Association for Computing Machinery*, 120–124. 10.1145/3288155.3288158

[CR44] Muros, R., Aragón, B. C., Y., & Bustos, J., A (2013). Youth’s usage of leisure time with video games and social networks. *Comunicar*, *40*, 31–39. 10.3916/C40-2013-02-03

[CR45] Organización Mundial de la Salud (2023). *Trastorno por uso de videojuegos. Clasificación Internacional de Enfermedades 11ª revisión. Estandarización mundial de diagnóstico en el ámbito de la salud*. https://www.who.int/standards/classifications/frequently-asked-questions/gaming-disorder

[CR46] Ortega-Jiménez, D., & Cedeño-González, G. & Marina del Rocío Ramírez Zhindón. (2023). [Uso Problemático De Videojuegos Y Flexibilidad De Afrontamiento en Adolescentes Ecuatorianos]. *Haaj*, 23(1). 10.21134/haaj.v23i1.748

[CR47] Plante, C. N., Gentile, D. A., Groves, C. L., Modlin, A., & Blanco, H., J (2019). Video games as coping mechanisms in the etiology of video game addiction. *Psychology of Popular Media Culture*, *8*(4), 385–394. 10.1037/ppm0000186

[CR49] Roslan, M. Z., Amran, M. S., & Sommer, W. (2024). Reliability and validity of the Game Addiction Scale in Malaysian Adolescents. *International Journal Of Adolescent Medicine And Health*, *36*(6), 571–578. 10.1515/ijamh-2024-0150 39582428 10.1515/ijamh-2024-0150

[CR50] Rosseel, Y. (2025). *The Lavaan tutorial*. https://lavaan.ugent.be/tutorial.pdf

[CR51] Saunders, J. B., Hao, W., Long, J., King, D. L., Mann, K., Fauth-Bühler, M., Rumpf, H. J., Bowden-Jones, H., Rahimi-Movaghar, A., Chung, T., Chan, E., Bahar, N., Achab, S., Lee, H. K., Potenza, M., Petry, N., Spritzer, D., Ambekar, A., Derevensky, J., Griffiths, M. D., Pontes, H. M., Kuss, D., Higuchi, S., Mihara, S., Assangangkornchai, S., Sharma, M., Kashef, A. E., Ip, P., Farrell, M., Scafato, E., Carragher, N., & Poznyak, V. (2017). Gaming disorder: Its delineation as an important condition for diagnosis, management, and prevention. *Journal of Behavioral Addictions*, *6*(3), 271–279. 10.1556/2006.6.2017.039 28816494 10.1556/2006.6.2017.039 PMC5700714

[CR52] Secretaría de Salud (1983). [Reglamento de la Ley General de Salud en Materia de Investigación para la Salud]. http://www.salud.gob.mx/unidades/cdi/nom/compi/rlgsmis.html

[CR53] Secretaría de Salud (2013). [Norma Oficial Mexicana NOM-012-SSA3-2012. Que establece los criterios para la ejecución de proyectos de investigación para la salud en seres humanos]. http://dof.gob.mx/nota_detalle.php?codigo=5284148&fecha=04/01/2013

[CR54] Svetina, D., Rutkowski, L., & Rutkowski, D. (2020). Multiple-Group Invariance with Categorical Outcomes Using Updated Guidelines: An Illustration Using M plus and the lavaan/semTools Packages. *Structural Equation Modeling: A Multidisciplinary Journal*, *27*(1), 111–130. 10.1080/10705511.2019.1602776

[CR55] Tereshchenko, S. Y., Gorbacheva, N. N. (2024), [ВАЛИДАЦИЯ, РУССКОЯЗЫЧНОЙ ВЕРСИИ ОПРОСНИКА, & ОЦЕНКИ ЗАВИСИМОСТИ ОТ КОМПЬЮТЕРНЫХ ИГР У ПОДРОСТКОВ (GAME ADDICTION SCALE FOR ADOLESCENTS)]. *Сибирский психологический журнал*. 93, 22–36. 10.17223/17267080/93/2

[CR56] Thorens, D. G., Achab, S., Rothen, S., Khazaal, Y., & Zullino, D. (2016). Addiction aux jeux vidéo, que du virtuel? *Revue Médicale Suisse*, *2*(531), 1554–1556. 28678449

[CR58] World Health Organization (2021). CIE-11. *Clasificación Internacional de Enfermedades, 11.a revisión. Estandarización mundial de la información de diagnóstico en el ámbito de la salud*. https://icd.who.int/es

[CR59] World Medical Association (2013). Declaración de Helsinki de la AMM. Principios éticos para las investigaciones médicas en seres humanos. https://www.wma.net/es/policies-post/declaracion-de-helsinki-de-la-amm-principios-eticos-para-las-investigaciones-medicas-en-seres-humanos/11859824

[CR57] World Health Organization. (2019). Sharpening the focus on gaming disorder. *Bulletin of the WHO*, *97*(6), 382–383. 10.2471/BLT.19.020619 10.2471/BLT.19.020619 PMC6560378 31210674

[CR60] Xu, L., Cai, Y., & Tu, D. (2019). Psychometric properties and structures of the IAT, GPIUS and gas scales: A bifactor approach. *Journal of Pacific Rim Psychology*, 13. Scopus. 10.1017/prp.2018.27

